# Therapeutic miRNA-Enriched Extracellular Vesicles: Current Approaches and Future Prospects

**DOI:** 10.3390/cells9102271

**Published:** 2020-10-11

**Authors:** Javaria Munir, Jeong Kyo Yoon, Seongho Ryu

**Affiliations:** 1 Soonchunhyang Institute of Medibioscience (SIMS), Soonchunhyang University, Cheonan, Chungcheongnam-do 31151, Korea; javaria_munir@live.com; 2 Department of Integrated Biomedicine, Soonchunhyang University, Cheonan, Chungcheongnam-do 31151, Korea; 3 Department of Biochemistry, Quaid-i-Azam University, Islamabad 45320, Pakistan

**Keywords:** extracellular vesicles, miRNA, therapy, drug-delivery, diseases

## Abstract

Extracellular vesicles (EVs) are 50–300 nm vesicles secreted by eukaryotic cells. They can carry cargo (including miRNA) from the donor cell to the recipient cell. miRNAs in EVs can change the translational profile of the recipient cell and modulate cellular morphology. This endogenous mechanism has attracted the attention of the drug-delivery community in the last few years. EVs can be enriched with exogenous therapeutic miRNAs and used for treatment of diseases by targeting pathological recipient cells. However, there are some obstacles that need to be addressed before introducing therapeutic miRNA-enriched EVs in clinics. Here, we focused on the progress in the field of therapeutic miRNA enriched EVs, highlighted important areas where research is needed, and discussed the potential to use them as therapeutic miRNA carriers in the future.

## 1. Introduction

The past few decades have seen enormous research in the field of extracellular vesicles (EVs). EVs produced by cells are divided into broad categories of exosomes, microvesicles, and apoptotic bodies. This categorization depends upon their origin in cell. This review mainly points out to ‘exosome’ related studies while referring to them as EVs (unless otherwise mentioned). Exosomes are small vesicles produced endogenously by almost all types of cells for intercellular communication. Their ability to carry their cargo, specifically nucleic acids, i.e., mRNA and miRNA to the recipient cells, has attracted the attention of the drug-delivery community [1,2]. In this paper, words such as ‘extracellular vesicles’, ‘exosomes’, ‘RNA’, ‘miRNA’, ‘treatment’, ‘non-coding RNA’, ‘therapy’, were used to search in “PubMed” and “google scholars”. Generally, articles from the last ten years were preferentially included.

Previously, synthetic liposomes and nanovesicles have been used to deliver therapeutic molecules, including miRNA, to cure certain diseases. However, the efforts to make them biocompatible and safe in every aspect have not been met with absolute success. In this regard, intrinsic EVs may provide immunologically safer options as carriers of therapeutic molecules, specifically miRNA [1,2]. Two important features for considering EVs as potential option for drug-delivery are their compositional properties and nano-mechanical properties. Compositional properties include their surface lipid/protein content and capacity to carry specific molecules in their lumen. Moreover, nano-mechanical properties include size and colloidal ability, which are important in EV-cell interaction. Compositional properties have been studied extensively; however, comparatively little attention has been given to nano-mechanical features until now. Therefore, this review only covers the aspect of compositional characteristics of EVs as a therapeutic carrier [3].

**Figure 1:**
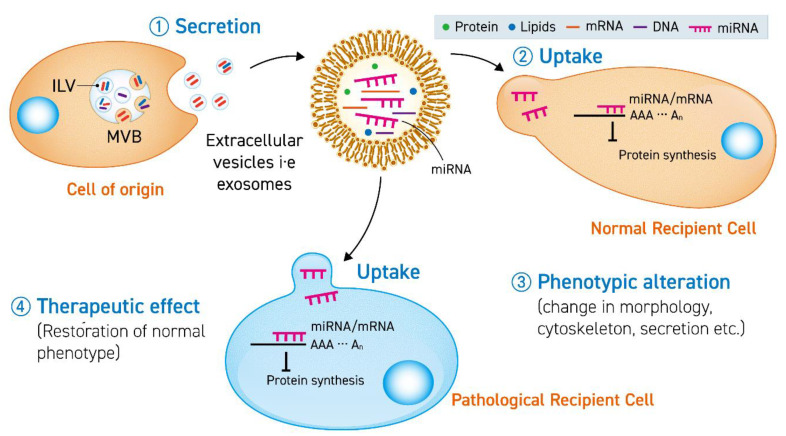
The secreted extracellular vesicle (EV) (1) carrying miRNA is uptaken by a normal cell (2) or pathological cell to cause a phenotypic change (3) or therapeutic effect (4) by translational inhibition of mRNA in the recipient cells. ILV stands for intraluminal vesicles and MVB stands for multivesicular bodies.

Studies with endogenous EVs have led to the identification of miRNAs in their cargo. These EVs can be used to restore normal phenotype in in-vitro diseased models (Figure 1). Exploiting this natural mechanism, efforts have been made to isolate EVs from cell media and body fluids to enrich them with therapeutic miRNAs. However, despite the rapid expansion of investigations regarding miRNA-enriched EVs, there have been only a few human clinical trials reported to date as evidence of treating pathologies. One of the reasons behind the lack of clinical studies is insufficient pre-clinical investigations in animals. The in-vivo studies, which are the precursor to human trials, should make sure the availability of: (1) higher yield of purified exosomes, (2) well-defined and optimized conditions to maintain exosome-secreting cells for reproducible results, (3) an optimized number of doses and quantity of variations of immune systems in subjects. Extensive efforts should be focused on developing and engineering EVs specifically for in-vivo administration.

Several technical limitations and gaps in our knowledge may become a possible hurdle for therapeutic miRNA enriched EVs to reach clinics. To overcome such obstacles, numerous methods to successfully obtain clinical-grade exosomes and manipulating them to target specific cells/tissues have been developed [4]. Moreover, engineering these EVs to load specific therapeutic miRNAs has achieved special attention in the EV-community and drug-delivery experts. Until recently, a number of investigational studies have focused on the endogenous mechanisms of miRNA loading/sorting in EVs, to pave the way of engineering miRNA-based EV carriers for alleviating diseases using in-vitro and in-vivo models. However, there is still an unsettled debate about whether the miRNAs are sorted/loaded actively or passively in endogenous systems. The factors responsible for determining the choice of active or passive loading have just begun to be studied. Uncovering this can provide important information to develop an improved protocol for engineering miRNA-enriched EV therapies in the future.

To the best of our knowledge, the subject of therapeutic miRNA-enriched EVs has not been extensively and exclusively reviewed previously. However, there have been some reviews summarizing the intrinsic sorting mechanisms of miRNA in exosomes [5]. Moreover, O’ Brien et al. published a review covering all non-coding/coding RNAs in EVs (including mRNA, tRNA fragments, snoRNA, ln-RNA) but the focus to miRNA is limited [6]. In this review, we will focus on EV based therapies with reference to enrichment of therapeutic miRNAs and their clinical applications. Additionally, the current gaps and challenges will be discussed in terms of potential clinical outcomes for therapeutic interventions in the future.

## 2. Principal Concepts about Therapeutic miRNA-Enriched EV

### 2.1. Extracellular Vesicles

Extracellular vesicles are small vesicles enclosed by phospholipid-bilayer, secreted by eukaryotic cells. They are found in body fluids, such as blood, tears, sweat, ascites, cerebrospinal fluid, etc. In laboratory conditions, they can be obtained from the culture media of cell lines [1,2]. The term ‘extracellular vesicles’ refer to a broad category of vesicles, which are further divided on the basis of their biogenesis pathway, i.e., exosomes, microvesicles, and apoptotic bodies. Exosomes are formed by inward budding of multivesicular bodies (MVBs). The size of microvesicles is variable, reaching up to 1 micrometer, produced by outward budding of the cell membrane, whereas apoptotic bodies are produced from the cell membrane’s fragmentation when cells undergo apoptosis. Usually, EVs within size range 50–300 nm are used for studies referring to them as ‘exosomes’ [7]. However, due to lack of sufficient biomarkers and overlap in size-range, it is difficult to discriminate between the types of vesicles.

Extracellular vesicles (EVs) carry biomolecules, such as mRNA, proteins, miRNA, metabolites, and have the ability to deliver them to cells along short and long distances. Blood can be used as a medium of transport to deliver EVs over long distances. Their ability to travel in blood and pour their content to recipient cells with functional integrity make them excellent candidates for drug-delivery. Moreover, due to Cluster of Differentiation-47 (CD-47) expression on the EV surface, they can avoid mononuclear phagocytic system in blood [8]. Another important aspect to consider EVs for a drug-carrying vehicle is their similar clearance rate to synthetic liposomes [9]. Synthetic liposomes may accumulate in the spleen and liver depending upon their surface ligands and composition. A similar strategy of surface modification can be applied to EVs to target specific tissues and avoid off-target accumulation [10]. Moreover, due to the intrinsic nature of EVs, they lack the risk of immune rejection as posed by synthetic liposomes. The ideal drug-delivery EV should be able to recognize particular recipient cells. EVs recognize recipient cell surface through their lipids and membrane proteins. The EV surface lipids and membrane proteins can be manipulated to engineer suitable EV-based therapies for special purposes. For example, EVs for neurodegenerative disorders should be optimized to cross the blood–brain barrier (BBB) by engineering them to recognize the recipient cell [11,12]. Secondly, EVs can be loaded with a variety of molecules. To the best of our knowledge, most studies about EV-based therapies utilize miRNA or siRNA as a therapeutic substance encapsulated in EV [13]. The endogenous machinery to sort miRNA can be exploited to manufacture EV-based miRNA therapies. However, there still remains a difficulty in identifying methods to obtain purified homogenous EVs, which can be used in actual clinical settings. In short, it is crucial to understand the benefits of EV-miRNA therapies over the synthetic systems and identify the key challenges that need to be overcome before these EV-miRNA therapies can be available commercially.

### 2.2. miRNA

miRNAs are small non-coding, single-stranded molecules, discovered for the first time in *Caenorhabditis elegans* in 1993 [14]. They are used for inhibiting translation by blocking mRNA or degradation of mRNA [15], based upon complementarity of mRNA-miRNA sequence. During miRNA biogenesis, RNA Pol II transcribes primary miRNA (~150 nt). Drosha cleaves primary-miRNA into precursor miRNA (~70 nt), which is exported to the cytosol. Dicer catalyzes precursor-miRNA into mature miRNA (~25 nt). Further, miRNA can be recruited by RNA-induced Silencing Complex (RISC) for silencing mRNA through binding to its 3′ UTR, 5′UTR, or intron [15]. The mechanism through which miRNA is able to silence mRNA is similar to siRNA-mediated silencing, which makes them good candidates for EV mediated therapies. miRNA is small in size and low in weight so it can be transfected efficiently in cells generally and in EVs specifically. Moreover, pathologies such as cancer, inflammation etc., exhibit alteration in the levels of miRNAs, either in upregulation or downregulation. Thus, administering exogenous miRNAs via EVs to restore the normal levels may alleviate the disorders. miRNAs were found as cargo (along with other proteins, lipids, RNA, etc.) in exosomes secreted from T cells to target immune-presenting cells, for influencing translation. This emphasized the presence of the physiological importance of miRNA in exosomes [16]. However, mechanistic details of packaging miRNA within EV remains elusive with a debate going on about whether the sorting is a passive or active phenomenon.

Since miRNAs can remain stable when they are encapsulated in EVs, they are able to travel long distance in body fluids, e.g., blood, without being degraded by extracellular nucleases. The miRNA remains intact functionally in the recipient cells. Therefore, it makes them a good candidate for EV-based therapies. The potential of these miRNA and related molecules are a big hope for future EV-based therapies, despite the fact that, currently, the problem of off-target effects and specific recognition of target mRNA, is still unresolved.

## 3. Progress in miRNA-Enriched EV Therapies

**Figure 2:**
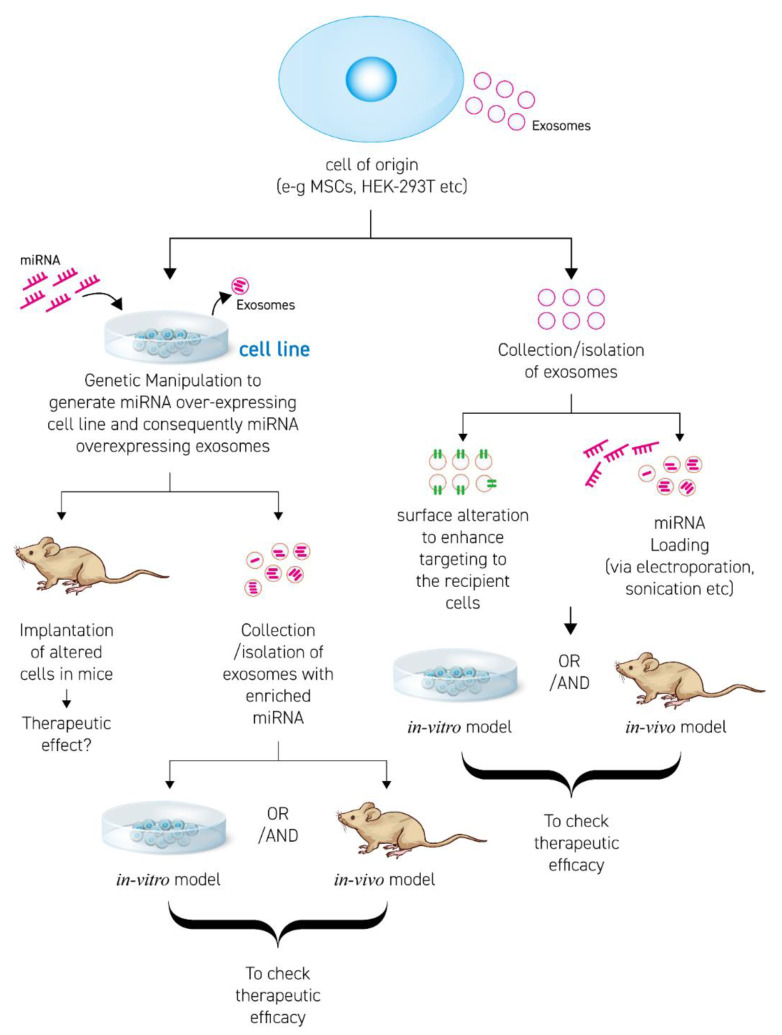
Scheme to develop and engineer therapeutic-miRNA EV based therapies.

Enrichment/loading of miRNA in EVs is accomplished by two approaches (Figure 2). The first approach is producing a cell line over-expressing (o/e) the desired therapeutic miRNA. The o/e cell line then exhibits a high concentration of miRNA in their cytosol, followed by EV secretion with enclosed therapeutic miRNA. The second approach is isolating EVs from source (cell line or body fluids) and loading them with the miRNA of choice by using chemical or physical methods/techniques. In Figure 2, some aspects of designing appropriate EV-based miRNA therapies are discussed.

### 3.1. Transfection

Since it is well established that enhancing concentration of miRNA in cytosol may increase their passive loading in EVs, it is possible to transfect miRNA of choice into cells to design EV-therapy. One of the prerequisites of transfection is the selection of appropriate cell type. Until now, Mesenchymal Stem Cells (MSCs) and Adipose derived stem cells (ADSCs) are used commonly as ‘biofactories’ to produce EV with loaded miRNA of choice [17].

MSCs can be obtained from various sources, e.g., bone marrow, adipose tissue, umbilical cord, etc. They are naturally involved in the local maintenance and homeostasis of these microenvironments. The tissue-source of MSCs determine the kind of exosomes obtained from it. Overall, MSCs are easy to isolate and expand in-vitro. Therefore, their secreted exosomes can be good candidates for therapeutic purpose [16]. An example is bone marrow derived MSC transfected with miR-29b for healing injured spinal cord in rats via exosomes encapsulated miR-29b [18].

However, it is difficult to use MSCs to obtain high yield of EVs for therapeutic purpose. Moreover, in personalized therapies, obtaining MSCs from aged subjects is difficult due to a lower number of MSCs in their bodies. This implies that the cell system should be chosen while keeping in mind the purpose of miRNA-loading, including the disease under consideration, the communication dynamics between EV-producing cells and the recipient cell, EV-secretion rate, and the ability of EVs to uptake exogenous therapeutic miRNAs [19].

Other cell systems using the same principle are developed for large yields. One example is represented by immortalized CEVEC’s amniocytic production line (CAP) cells. CAP cells were able to produce functional exosomes, and were also able to combine active and passive mechanisms of miRNA loading for EV-enrichment (miR-493 and miR-744). Both contained motifs in 3p strand, which helped them to load in EVs. Further, they can unload their cargo in ovarian cancer cells (SKOV3), thus, down-regulating mRNA targets [20]. Additionally, the immortalized cell line from tumor cells can provide high yield of EVs, but cannot be used for therapeutic purpose due to safety issues [19].

Kojima et al. has reported HEK-293T and a human mesenchymal stem cell system that exploits exosome biogenesis, RNA packaging, secretion, targeting, delivering into cytosol used for treating Parkinson’s disease in in-vitro and in-vivo models. However, the system was used to assess functional mRNA delivery rather than miRNA [21]. Improvement in these cell systems may provide options for transfecting cells to secrete therapeutic-miRNA enriched EVs.

Electroporation is also used for transfection to introduce miRNA in EVs. The process involves using voltage and pulse to open up pores in EVs so that nucleic acids, such as miRNA, can get entry to vesicles, followed by healing of these pores [22]. The miRNA-enriched EVs can be used to target the recipient cells to cure diseases. However, EVs from different sources require different voltage/pulse due to different membrane protein composition. Moreover, there are some problems to the process of electroporation: (i) contamination of reagent [8], (ii) aggregation of EVs [23], (iii) aggregation of miRNA [23], (iv) leakage of endogenous cargo when pores in EVs open. Leakage of endogenous cargo, including miRNA and proteins during electroporation, may cause functional abnormalities when the cargo is delivered to the recipient cells. Nevertheless, the need of specialized equipment makes it difficult to perform. Pomatto et al. addressed the problems by introducing cel-39, miR-451a and miR-31-5p in human plasma-derived EVs through electroporation, followed by successful functional delivery in tumor endothelial cells (TEC). These miRNAs remained functional in hepatocellular carcinoma cell lines and induced apoptosis [24]. In addition to small RNAs (including siRNA and miRNA), miRNA mimics and miRNA inhibitors can be loaded in EVs via electroporation. There are chances that the yield of miRNA loaded in EVs would be over-estimated because of aggregation. Future technical improvements are required to provide solutions to the problems stated and refine the technique for future interventions [25].

### 3.2. Surface Modification and Membrane Proteins

Proteins on the surface of exosomes are generally representative of parental cells. These proteins include tetraspanins (CD-81, -82, -37, and CD-63), membrane trafficking proteins, cytoskeletal proteins, and two members of Endosomal Sorting Complex Required for Transport (ESCRT) pathway, i.e., Alix and TSG-101 (Tumor Susceptibility Gene 101). The proteins have limited tendency to target specific tissue. Moreover, these proteins allow exosomes to intrinsically accumulate in the liver, kidney, and spleen. Further, they can be excreted out via bile, renal filtration, and reticuloendothelial phagocytosis [26]. Therefore, for enhancing specific targeting and decreasing the clearance rate, it is highly recommended to modify the surface of exosomes. This is possible by modifying surface membrane proteins by direct methods or by genetic alteration of exosome membrane proteins.

Direct modification of exosome surface may occur via non-covalent or covalent methods. The non-covalent method involves mixing protein with exosomes. On the other hand, the covalent method requires attaching a peptide with covalent linkage. Studies have shown covalent attachment of the c(RGDyK) peptide on the exosome surface for specifically targeting avb3 on the cerebral vascular endothelial cell to suppress apoptosis and inflammatory response in ischemia, and deliver curcumin encapsulated as cargo [27]. However, the questions of how valid are these techniques for designing targeted therapies as miRNA-enriched EVs is open for further investigation. Direct methods do not require sophisticated purification. However, both direct methods (i.e., covalent and non-covalent) may have chemical contamination and variable efficiency of modification. Moreover, non-covalent attachment may undergo detachment in physiological conditions [28].

Genetic alteration involves modulating cells to produce a particular protein on the exosome surface. For example, HEK293 cells are genetically altered to produce GE11 peptide (YHWYGYTPQNVI) over-expressed on the secreted exosomes. GE11 is less mitogenic than EGF, therefore they are clinically safer to target cells with EGFR rather than EGF. These GE11 expressing exosomes can bind to EGFR on EGFR-positive breast cancer cells in mice, delivering their let-7 cargo as treatment strategy [29]. Bellavia et al. prepared exosomes from HEK293 cells carrying BCR-ABL siRNA and expressing IL-3L on their surface. These IL-3L expressing exosomes can target IL3-R expressing blast cells in Chronic Myelogenous Leukemia (CML) to induce recovery of CML [30]. Genetic modification produces more uniform population and stable target specificity. It is more expensive than a direct method. Furthermore, it raises safety concerns and, thus, is a disadvantage for clinical applications [29].

### 3.3. RNA Binding Proteins (RBPs)

miRNA sorting in exosomes of endogenous systems is facilitated by RNA binding proteins. RBP can directly bind to miRNA via RBP-recognizing sequences or via unidentified mechanisms. For example, hnRNPA2B1 recognizes GGAG/UGCA motif in miR-198 and miR-601 to sort them in exosomes. Silencing of this protein decreases miRNA in exosomes by 13%, demonstrating its positive regulatory role in exosomal sorting of miR-198 and miR-601. Moreover, it is important to consider the post-translational modification of RBP, e.g., sumoylation [31,32]. Another RBP, i.e., SYNCRIP, associates with miR-3470 and miR-194-2-3p via GGCU, and sorts them in exosomes. Its silencing via shRNA led to decrease in miRNA concentration. SYNCRIP association with miRNAs is ensured via its domain namely, N-terminal unit for RNA Recognition (NURR). Removal of this N-terminus domain hinders miRNA binding and exosomal loading [33]. In addition, RBPs, such as hnRNPH1, might be involved in negatively regulating entry of miRNA in exosomes. Its silencing led to increase in exosomal RNA, leading to the hypothesis that hnRNPH1 may be involved in sequestering miRNA in cytosol [34]. Moreover, indirect recognition of miRNAs by RBP takes place via intermediate protein. An example is adaptor related protein complex-2 mediated interaction of MEX3C and miRNA-451a [34]. Sometimes, more than one RBP are involved in sorting miRNA. For instance caveolin-1 assists sorting of miRNA along with hnRNPA2B1 [35].

Methods used to identify RNA-binding proteins from exosomes may miss some RBPs because of their low quantity in exosomes or due to binding to very few miRNAs. Moreover, systems used to identify RBP (i.e., knock-down and over-expression) cannot distinguish between primary and indirect role of RBP in miRNA sorting. To overcome this problem, Shurtleff et al. have demonstrated the use of cell-free system to understand the role of YBX-1 to sort miRNA-223 [36]. Despite the presence of gaps in our knowledge about function of RBPs, it is speculated that these proteins are involved in maintenance of RNA in exosome or detached to target other RNA in recipient cells. However, the mechanistic details are not clear. Further studies are required to integrate all of these concepts about RBP mediated loading of miRNA in exosomes. This will pave the way for utilization of RNA binding proteins in developing and improving miRNA-enriched EV therapies.

## 4. Important Considerations for miRNA-Enriched EV Based Therapies

### 4.1. Stoichiometry

The ability of miRNA-enriched EVs to treat diseases depends upon the quantity of miRNA required to inhibit mRNA targets in the recipient cells. Most of the studies, if not all, rely on in-vitro evidence, in which an excess of exosomes is used [37]. This raises a question of the relevance of these studies in the physiological context. Studies of exosomal miRNAs from different sources, including plasma, seminal fluid, dendritic cells, mast cells, and ovarian cancer cells, have found that there is less than one given miRNA per exosome [37]. Given that exosomal uptake is selective [38], the physiological relevance can only be possible if at least a small percentage of exosomes in the total exosome population contain a very high concentration of a particular miRNA [37]. In human neural stem cells, in which miR-1246 is the most abundant miRNA in exosomes, 10 copies of miR-1246 should be present per exosome to be physiologically relevant [39]. Moreover, in-vitro experiments using HeLa cells showed a functional transfer of miR-1246. This was confirmed by miR-1246/FAM53C in-vitro reporter luciferase assay in HeLa cells exhibiting protein reduction by targeting 3′UTR FAM53C after administering hNSC exosomal preparations [40].

To further understand the function of exosomal miRNAs in diseases, it is important to quantify miRNAs poured in each and every recipient cell. Lee et al. [32] showed that 10 μg of epithelial microvesicles (~10^10^ of miRNA-17/93) was sufficient to activate 10^5^ macrophages in-vitro and in-vivo. The same strategy as applied in microvesicles can probably be applied to exosomes. However, the evidence of the quantity of miRNA in a defined quantity of exosome in any system is lacking. Moreover, a variety of miRNAs can be contained in exosomes, each targeting a different mRNA and thus a different signaling pathway within a cell. The phenotypic effect observed in recipient cells after an entry of exosomal miRNAs is the result of collective action of all these miRNAs. Thus, it is difficult to completely and precisely understand the effects of exosomal miRNAs in target cells. There is a need to develop techniques for more in-depth and precise analysis of exosomal miRNAs [40]. Before using therapeutic miRNA enriched EVs in clinics, the dosage and quantity of EVs, as well as quantity of functionally relevant miRNA to target mRNA in recipient cells in diseased tissue, should be precisely calculated. Usually, EVs are present as a heterogenous population, including protein aggregates, non-EV vesicles of similar size, and other RNA molecules, which raises concerns about false positive signals. Quantification takes place in the units of mass or number in a given sample. However, there is no universal standard for EV and miRNA quantification and, hence, it becomes difficult to compare two independent studies, which emphasizes the need of appropriate controls [41]. Developing precise methods universally accepted by the scientific community for quantification of EV will pave the way for future advancement in this field.

### 4.2. Storage and Stability

One of the challenges that exists in the use of developing therapies based on exosomal miRNAs is the stability. The following passages will highlight the studies demonstrating stability of the isolated EVs for therapeutic miRNA enrichment, stability of EVs after the enrichment of miRNA, and stability of EV-miRNA in physiological conditions. All of these aspects are crucial in determining the feasibility of using therapeutic miRNA in translational medicine.

EVs can be obtained from different sources, including body fluids and conditioned media. Blood serum can be stored at −80 °C before EV isolation for downstream applications. Moreover, the International Society of EVs has recommended phosphate buffer saline, stored in a siliconized vessel at −80 °C for avoiding possibility of adherence to walls [42]. Jeyaram et al. have reported successful isolation of EVs maintained their morphology, RNA content, integrity, etc., after 30 years storage at −80 °C. This shows how long term stored samples can be harnessed to develop EV-based therapies [43].

Bovine milk derived EVs are generally used as a drug carrier, such as paclitaxel. These EVs remained stable with paclitaxel for four weeks stored at −80 °C. As far as storage and stability of miRNA-enriched EVs is concerned, there are limited specific studies to exhibit how long can they can be stored with complete integrity and miRNA activity before testing in in-vitro or in-vivo models [42]. However, Coennan-Stas et al. focused on determining the stability of EV-miRNA in physiological conditions. They found that EV-miRNA can remain stable in physiological environment for 1.5–13 hrs. Additionally, on the basis of results obtained from different miRNA (let-7, miR-1, miR-206, miR-233 and miR-133a), it was concluded that the intrinsic sequence of miRNA determines their stability. There exists a positive co-relation of stability of miRNA with GC content. For example, miR133a is the most stable due to the highest GC content among all the miRNAs studied [44]. Despite the efforts to understand stability issues, there is a need to develop universally accepted guidelines before introducing EV-based therapies in clinics.

## 5. Disease Treatment Using miRNA-Enriched EVs

### 5.1. Neuroprotection

Since 98% of the drug molecules have limitation to cross the blood–brain barrier (BBB) [9], it is important to discover innovative solutions to overcome the obstacle. In this regard, EVs may provide efficient therapies, which may be able to cross the BBB. Exosomes enriched in miR-30d target Beclin and Atg5 in models of brain injury. Beclin and Atg5 are autophagy-associated proteins. The decrease in the levels of these proteins lead to alleviation of cerebral injury by promoting microglial polarization and suppressing autophagy [45]. Moreover, exosomes enriched with miR-133b can initiate recovery of stroke [46]. One of the most common methods to develop exosomes enriched with therapeutic miRNA is transfecting adipose tissue derived stem cells (ADSCs) or mesenchymal stem cells (MSCs) with the miRNA under consideration. However, brain extract from a mouse with Traumatic Brain Injury (TBI) can be used to influence microglial cells to secrete miR-124-3p enriched exosomes. These miR-124-3p enriched exosomes can treat TBI by targeting focal adhesion kinase family-interacting protein (FIP200) [47].

Moreover, neuronal abnormalities characterized by dendritic morphology of neurons may be controlled by miR-26-5p enriched exosomes from astrocytes. Astrocytes are a type of glial cells required for synaptic transmission and nerve repair. Luarte et al. [48] identified that astrocyte-derived EVs can carry miR-26a-5p to the hippocampus neurons to modulate dendritic arborization. However, there is a need to study which factors can cause loading of miR-26 in EVs secreted by astrocytes. Such studies may pave a way to treat schizophrenia, depression, and bipolar disease in the future.

### 5.2. Cardiac Diseases

Acute myocardial ischemia (AMI) is one of the leading causes of death across the globe. Attempts have been made to elucidate the role of miR-126 enriched exosomes from adipose stem cell derived cells (ADSCs) to treat AMI. Luo et al. has developed an in-vitro model of myocardial infarction and treated the cell lines with miR-126 enriched exosomes obtained from ADSCs over-expressing miR126 [49]. The results showed reduction in cell injury, inflammatory factor and fibrosis related protein in cells. Intravenous delivery of miRNA-126 enriched exosomes to rats suffering from myocardial infarction also exhibited signs of reduced myocardial injury, reduced fibrosis, and decrease in inflammatory cytokine expression. Na Wang et al. described that angiogenesis can be triggered in heart which has experienced myocardial infarction. For initiating angiogenesis, miR-210 enriched EVs from MSC can be used to target Efna-3 in endothelial cells. siRNA was used to decrease the expression of miR210 in MSC. Hence, MSC-derived EVs lacked miRNA-210 and result in less angiogenesis. This suggests how miR-210 enriched EVs and miR-126 EVs can be used as future treatments after heart attacks [50].

The use of miRNA-enriched EVs to treat myocardial infarction (MI) presents important questions of mode of delivery. Song et al. used direct intramyocardial injection of miR-21 enriched EVs to initiate anti-apoptotic effect in heart. The direct injection may increase the possibility for EV-miR-21 to be uptaken by other neighboring cells. This problem may be solved by adjusting a low dose or by manipulating the surface of EVs so that they can only target cardiomyocytes. Moreover, miR-21 is pleiotropic and has pro-fibrogenic effect. To avoid the pro-fibrogenic side-effect, miR-21 can be loaded in combination with some other miRNA or a drug molecule in EVs, which can nullify the harmful effect of miR-21, leaving only its cardioprotective effect intact [51]. Loading EVs with an optimized combination of miRNAs to have a therapeutic effect and avoid undesirable collateral damage demonstrates challenges and precise clinical parameters which need further investigation.

In addition to MI, cardiac dysfunction can arise as a secondary cause of phenomena such as sepsis. Sepsis combined with cardiac abnormalities can ultimately lead to death. Exosomes derived from MSC are enriched in miRNA-223, which can be used as cardio-protective agents for cardiomyocytes. Wang et al. demonstrated the significance of miR-223 enriched exosomes via knock-out systems. Exosomes obtained from MSC KO miR-223 cannot alleviate inflammation in cardiac injuries in animal models. Moreover, it has been shown that miR-223 enriched MSC derived exosomes enter cardiomyocytes and decrease Sema3A and Stat3 expression. The reverse is true for miR-223 KO exosomes, which result in increased expression of Sema3A and Stat3. Additionally, miR-223 lacking exosomes did not exert protective effect on cardiac dysfunction and inflammation [52]. This suggests how therapeutic miRNA in EVs can serve as treatments for the future (Table 1).

### 5.3. Cancer

Cancer progression and metastasis in different types of cancers (e.g., pancreatic cancer, endometrial cancer, and glioblastomas) are associated with downregulation of tumor suppressor miRNAs. Restoration of these tumor suppressor miRNAs as EV-based therapies may lead to tumor inhibition by reducing proliferation and invasion (Table 1). The mode of delivery is crucial to determine the dose and frequency of therapy. Direct intra-tumor injection of miR-335 enriched EV led to cancer inhibition in hepatocellular carcinoma in xenograft models by reducing proliferation and increasing apoptosis. Moreover, qRT-PCR analysis demonstrated downregulation of thirteen genes including Casein kinase 1 gamma 2 (CSNK1G2) and human zinc finger MYND-type containing 8 (ZMYNO8), which are known to have differential expression in early cancer and metastasis. However, the direct evidence of EV-encapsulated miR-335 targeting CSNK1G2 and ZMYNO8 in hepatic cancer is lacking [67,75]. To treat cancers, EVs can be obtained from different sources, such as fibroblasts [76], stromal cells [64], and umbilical cord stem cells [65] for further enriching them with therapeutic miRNA. Patient specific stem cells can be transfected with therapeutic miRNA, such as miR-302-367. These stem cells can be implanted in the brain of immunodeficient mice for continuous supply of EVs-enriched with miR-302-367 for the treatment of brain injury. The advantage of this kind of approach is a long term supply of exosomes from cells rather than a temporary supply of exosomes. This constant supply of exosomes suppresses tumor growth and development. Moreover, it also inhibits tumor initiation and tumor relapse. Such a strategy is beneficial for patients when surgical removal of tumor is not possible [40]. Although, studies, such as Fareh et al. [40], utilize animal models to understand the effect of therapeutic miRNA loaded EVs in cancer, most of the studies utilize cell monolayer cultures. Therefore, it is difficult to extrapolate the results to heterogenous tumors. Further studies are required to integrate the information about heterogeneity of tumors and response of stromal cells to EV-based miRNA therapies

### 5.4. Musculoskeletal Diseases

Musculoskeletal diseases include disorders of the bone, cartilage, and muscles, for example osteoarthritis (OA), which is the most common joint disease. Moreover, chondrosarcoma is the second most common cancer of musculoskeletal apparatus [74,76]. Previously, MSC-based therapy was used to regenerate bone/cartilage. However, it is difficult to trigger regeneration of cartilage because of it being inaccessible by cells. Therefore, exosomes provide therapeutic solutions. Exosomes from MSCs can induce osteoblast differentiation and muscle regeneration. miR-92-a-3p in MSC-derived exosomes may increase chondrogenesis and suppress cartilage degeneration via wnt5A, thus, can serve as treatment for OA [70]. Moreover, ADSC-derived exosomes carrying miR-375 target 3′UTR of insulin like growth factor binding protein 3 in bone marrow-derived stem cells in vitro to promote bone formation which may serve as a good therapeutic option for calvarial defects [71]. However, in-depth investigation is required to apply these therapies in in-vivo models and humans.

## 6. Clinical Usage

EVs produced naturally from cells cannot be used directly for clinical uses. Good Manufacturing practice (GMP) is required for large-scale production of EVs. GMP takes into account several factors including cell-type, culture condition, type of culture media and cultivation vessel [4]. Five cell types, which have been used to produce EVs following GMP, are human cardiac progenitor cells, bone marrow MSC, adipose derived stem cells, monocyte derived dendritic cells (DCs), and HEK293 [4]. Clinical trials using DC-derived EVs started to appear on the scene after 2013, followed by clinical trials involving MSC-derived EVs [4]. Indeed, clinical trial of MSC-derived exosomes is the only trial, to the best of our knowledge, which carries KrasG12 siRNA for the treatment of pancreatic cancer. MSC is a well-established cell-bank for obtaining GMP-based EVs. Mendt et al. used bone marrow derived stem cells derived EVs to target oncogenic Kras via loaded siRNA in pancreatic cancer using in-vitro and in-vivo models, but did not reach human level trial [76,77]. In contrast, studies showed DC-derived EVs containing tumor antigen may cause inflammation. Although, MSC-derived EVs are loaded with siRNA and not miRNAs, it is likely to use these studies as a precursor to develop strategies for therapeutic miRNA enrichment of EVs. However, to overcome the limitation of insufficient production of natural EVs, extrusion of cells through a series of ultrafiltration lead to the production of extracellular vesicle mimetics (EVMs). It is worth noticing that the same number of cells can provide 20–100 fold more EVM than natural EVs [78]. In addition to human cell-derived EVs, EVs can be obtained from plants, referred to as plant-derived EVs [79]. However, plant derived EVs are still under-studied in clinics compared to human-derived EVs. Although GMP ensures obtaining clinical-grade EVs, which can be loaded with RNAs, the problem of ultra-purification for human interventional studies still persist. Moreover, batch-to-batch variation pose an obstacle for human studies [71]. Future studies are required to develop appropriate protocols for miRNA-enriched EVs.

## 7. Opportunities and Limitations

The field of miRNA-enriched EV therapies is expanding with the use of MSC as a cell source for EVs in preclinical and clinical trials. Moreover, with the increase in knowledge about EV-biology and technological advancement of precise methods, there is a possibility for treating disease symptoms including stroke, cardiovascular complications, etc.

Even though there are potential opportunities for miRNA-enriched EVs to alleviate diseases, there are some obstacles that are actively being studied. These include the methodological limitations to influence loading efficiency of miRNA in EVs. Moreover, there are limited studies regarding mass production of EVs. A mutual consensus is yet to be formed on standardized protocol for large-scale production and isolation of clinical-grade EVs. This may include modulating the EV biogenesis to yield high EV secretion [19,80]. A detailed criterion for characterization of EV is required, specifying the features of EVs suitable and safe for clinical usage. The choice of parent cell as a source of EVs has to be determined for engineering therapies best suited for physiological environment [71]. Moreover, the route of administration of EVs is also important, taking in consideration how long EVs can remain functionally stable in body fluids. Furthermore, the dose and other pharmacokinetics need to be assessed in detail before treatments are made open for public use.

## 8. Conclusions

EVs can be enriched with specific therapeutic miRNA for alleviating pathological cellular states in different diseases. However, the drug-delivery community and EV-scientists are still trying to develop precise ways to deal with clinical applications. Moreover, methods to commercially isolate EVs on a large-scale, methods to load miRNA in EVs, and safely deliver them to target tissues, remain areas of great interest for future studies. Investigating endogenous mechanisms of miRNA-sorting may open a new avenue in therapeutic interventions.

## Figures and Tables

**Table 1:** Therapeutic miRNA-enriched EVs: Evidence from Disease models.

	References	Therapeutic miRNA	Enrichment/Sorting Method of miRNAin EVs	Source of EVs	Disease Model	Mode of Action
**1.**	[49]	miR-126	Transfection of miR-126 mimic	Adipocyte-derived stem cells	Myocardial Infarction	Decrease in cardiac apoptosis and fibrosis
**2.**	[53,54]	miR-182-5p	Transfection with miR-182-5p	Mesenchymal Stem cells	Ischemic injury	Reduced apoptosis in cardiomyocytes
**3.**	[52]	miR-223	miR-223 KO	Mesenchymal Stem cells	Cardiac injury (sepsis)	Decreased inflammation and cell death
**4.**	[55]	miR-125-b	-	Mesenchymal Stem cells	Myocardial infarction	Reduced autophagy flux and cell death
**5.**	[51]	miR-21	Over-expression of miR-21	HEK-293T	Myocardial Infarction	Antiapoptotic effect by targeting PDCD4 in cardiomyoblasts
**6.**	[50]	miR-210	Over-expression of miR-210	Mesenchymal stem cells	Myocardial infarction	Initiation of angiogenesis by targeting EFna-3 in endothelial cells
**7.**	[40]	miR-1, miR-302-367	Over-expression of respective miRNAs	Patient-specific glioblastoma stem cells	Glioblastomas	Reduced Growth and invasiveness in xenografts
**8.**	[56]	miR-133	Transfection of MSCs with miR-133 mimic	Mesenchymal Stem cells	Intracerebral hemorrhage	Decrease in the number of neurodegenerative neurons
**9.**	[45]	miR-30d	Transfection of miR-30d	Adipose derived stem cells	Acute Ischemic Stroke	Microglial/Macrophage polarization and suppression of autophagy
**10.**	[48]	miR-26-5p	-	Astrocytes	-	Dendritic Arborization in hippocampus neurons
**11.**	[57]	miR-146a	Transfection with miR-146a mimic	Dendritic cells	Myasthenia Gravis	Altered T helper cells from Th1/Th17 to Th2/Treg
**12.**	[58]	miR-124-3p	Transfection with miR-124	Microglial	Traumatic brain injury	Inhibit neural autophagy
**13.**	[59]	miR-133	Transfection with miR-133 mimic	Mesenchymal Stem cells	Spinal cord injury	Regeneration of axons, preservation of neurons
**14.**	[60]	miR-124	Stable over-expression of the respective miRNA	HEK-293 cells	Huntington Disease	Reduction in RE1-Silencing Transcription Factor but no significant difference in behavior
**15.**	[46]	miR-133b	Knock-in and knock-down in miR-133b	Mesenchymal Stem cells	Stroke	Neurite Remodeling and post-stroke functional recovery
**16.**	[61]	miR-146-b	Transfection of the respective miRNA via electroporation	Mesenchymal Stem cells	Glioma	Reduced growth
**17.**	[62]	Anti-miR-142-3p	Transfection of the respective anti-miRNA via electroporation	Mesenchymal Stem cells	Breast Cancer	Reduced growth and metastasis
**18.**	[63]	miR-128-3p	Electroporation in exosomes	Dendritic cells	Colon Cancer	Increased Chemosensitivity
**19.**	[64]	miR-451a	-	Tumor-associated stromal cells	Pancreatic cancer	Promotion of apoptosis and reduced proliferation
**20.**	[65]	miR-302a	Over-expression of miRNA	Human Umbilical cord mesenchymal stem cells	Endometrial cancer	Reduced Proliferation and migration by targeting cyclin-D1
**21.**	[66]	miR-159	Incubation at 37 °C	Human Monocyte macrophage cells	Triple Negative Breast Cancer	Anti-cancer effect by targeting TCF gene
**22.**	[67]	miR-335	Transfection of exosomes	LX2 cells	Hepatocellular carcinoma	Less proliferation and enhanced apoptosis in hepatic tumors
**23.**	[68]	miR-181-5p	Over-expression of miRNA	Mesenchymal stem cells	Fibrosis	Reduces autophagy and inflammation by targeting and downregulating STAT-3 and BCl-2
**24.**	[69]	miR-185	Over-expression of miRNA	Mesenchymal stem cells	Oral Leukoplakia	Promote apoptosis by specifically targeting Akt genes
**25.**	[70]	miR-92a-3p	Over-expression of miRNA	Mesenchymal stem cells	Chondrogenesis (can be used in osteoarthritis)	Cartilage proliferation
**26.**	[71]	miR-375	Over-expression of miRNA	Mesenchymal stem cells	Rat model of calvarial defect	Bone regeneration
**27.**	[72,73]	miR-223	Over-expression of miRNA	Bone marrow derived stem cells	Experimental autoimmune hepatitis	Protection from liver injury
**28.**	[74]	miR-140	Over-expression of miRNA	Human Synovial stem cells	Osteoarthritis	Prevention of osteoarthritis by increasing proliferation and migration of chondrocytes
